# Tracking the Evolution of HIV/AIDS in China from 1989–2009 to Inform Future Prevention and Control Efforts

**DOI:** 10.1371/journal.pone.0025671

**Published:** 2011-10-05

**Authors:** Zhongwei Jia, Lu Wang, Ray Y. Chen, Dongmin Li, Lan Wang, Qianqian Qin, Zhengwei Ding, Guowei Ding, Chunpeng Zang, Ning Wang

**Affiliations:** 1 National Institute on Drug Dependence, Peking University, Beijing, China; 2 National Center for AIDS/STD Control and Prevention, China CDC, Beijing, China; 3 National Institute of Allergy and Infectious Diseases, U.S. National Institutes of Health, based at the U.S. Embassy, Beijing, China; Center for Complex Networks and Systems Research, Indiana University at Bloomington, United States of America

## Abstract

**Background:**

To determine policy implications, this analysis tracks the evolution of HIV/AIDS infection across China to understand current trends and potential risk factors.

**Methods and Principal Findings:**

A retrospective study with spatial analytical model and multilevel spatial models was conducted among 326,157 HIV/AIDS cases reported from 1989–2009. The results indicate that the distribution of HIV/AIDS was clustered at the county level with different directional distributions across China from 2003 to 2009. Compared to 2003, by 2009 there was a 122% increase in HIV cases among rural residents, 294% increase among urban residents, 211% increase among migrants, and 237% increase among permanent residents. The overall proportion of HIV by different routes of transmission showed dramatic changes with a 504% increase in sexual transmission of HIV, 90% decrease in blood/plasma transmission, and 35% decrease in injecting drug user transmission. Sexual transmission was the major transmission route among women (44%) and the elderly (59% in men, 44% in women) as well as among permanent (36%) and urban residents (33%). Among those <65 years old, women increased more than men, but among those ≥65 years, men increased more than women. Migrants contributed to the variance of HIV infection between counties but not within counties. The length of highway and urbanization combined with illiteracy were risk factors for HIV/AIDS.

**Conclusions/Significance:**

Rates of HIV/AIDS among permanent urban residents, particularly women and elderly men, have increased significantly in recent years. To prevent HIV from spreading further among the general population, additional attention should be paid to these populations as well as to migrants.

## Introduction

The first HIV infected patient in China was identified in 1985 in a foreigner at the Peking Union Medical College [1]. From 1985–1988, all identified cases, except for four due to contaminated, imported Factor VIII, and were infected overseas. From 1989–1994, most detected cases were clustered in Yunnan province among injecting drug users [1]. After 1995, however, with increasing market economy and more and more migrant workers, HIV spread across the country [2].

The concept of “migrant worker” results from the *hukou* residence system in China. All Chinese nationals are assigned a *hukou* at birth, which is the place of residence of his/her parents. Before 1980, the *hukou* system restricted movement from one place to another. People could travel but had no access to jobs, public services, education, or sometimes even food while away from their *hukou* location. However, with the reform of the Chinese economy starting in 1980, *hukou* became less restrictive which led to millions of farmers leaving their homes to seek better jobs and a more prosperous life in cities [3].

While away from home, families, and more traditional cultural values, men often work in construction, mines, or as long-distance truck drivers, and may seek sexual services. Women often work in the service industry and may even provide paid sexual services as sex workers. Some previous studies suggested that migrants have contributed to the HIV/AIDS epidemic [4]–[7].

In addition to the change towards a market economy, another significant shift in Chinese society over the past 25 years occurred in the area of cultural values and freedoms. The Chinese people enjoy much greater freedoms today in thought, speech, and choices, including sexual, than in years past. One consequence of this is a sexual revolution [8]–[9], with sexual transmission now being the dominant route of HIV spread in China [10].

This study assesses the effects of migrants, socioeconomic status, and social culture on the national changes of HIV/AIDS from 1989–2009. These results have implications for HIV/AIDS control policies in China and other countries because of the increase in globalization.

## Methods

### Design

**Figure 1:**
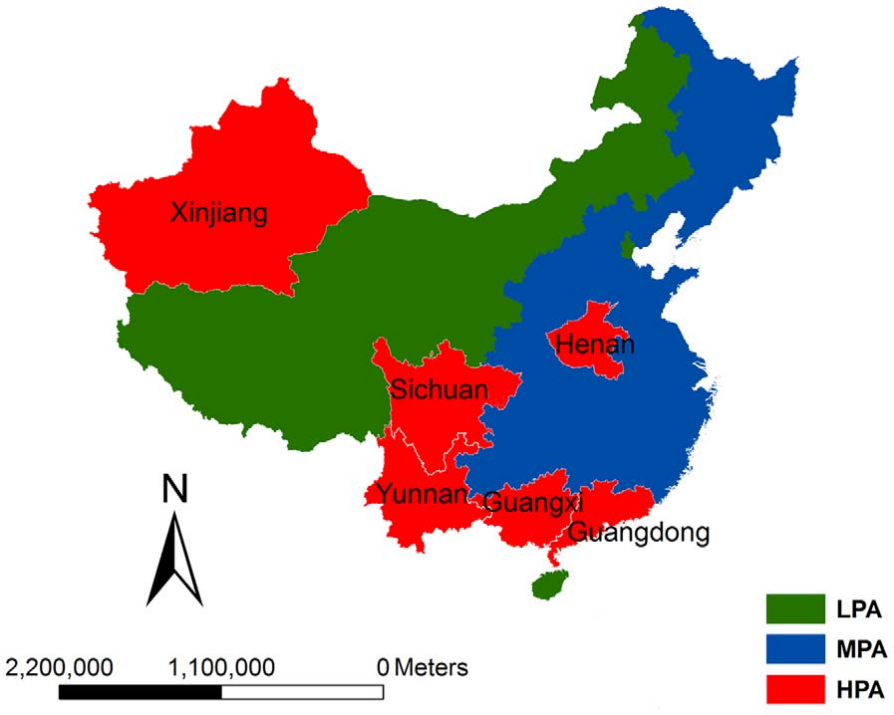
The geographic distribution of high, middle and low HIV/AIDS prevalence areas, according to the cumulatively reported numbers of reported HIV/AIDS cases in each province.

A retrospective study was used to analyse national level changes in the geography and demography of reported HIV/AIDS cases across China. The changes were assessed by high prevalence areas (HPA, includes 6/31 provinces [Guangdong, Guangxi, Henan, Sichuan, Xinjiang, and Yunnan] cumulatively reporting ≥100,000 HIV/AIDS cases), middle prevalence areas (MPA, includes 18/31 provinces cumulatively reporting 1000–99,999 HIV/AIDS cases) and low prevalence areas (LPA, includes 7/31 provinces cumulatively reporting <1000 HIV/AIDS cases) (Figure 1) ; by reported rural vs. urban location; and by Chinese residence permit location (*hukou*).

The China Ministry of Health scaled up the national notifiable disease reporting system for infectious diseases, including HIV/AIDS, in 2003 and began collecting significantly more case information for reported cases. Consequently, the analyses of HIV/AIDS distribution from 1989–2002 are more general whereas from 2003–2009 are more detailed.

### Data management

All HIV/AIDS cases recorded in the National AIDS/STD Surveillance System (NAIDSSS) at the Chinese Center for Disease Control and Prevention (China CDC) from 1 January 1989 to 31 December 2009 were analysed. All reported cases were checked and confirmed by local CDCs in county, provincial and national levels, respectively, before being recorded in the surveillance system to guarantee data quality. Demographic and socioeconomic data, such as migrant rates, income, and literacy rates, were obtained from census data provided by the National Bureau of Statistics of China and the School of Economics and Business Administration, Chongqing University [11]. Electronic maps with county boundaries were obtained from the Ministry of Water Resources. ArcGIS 9.3 software (ESRI Inc., Redlands, CA, USA) was used to create maps at 1∶100,000 scale.

### Ethical issues

This study focused on population-level analyses only and did not access any individually identifiable patient data. Because of this, ethics committee approval was not sought.

### Spatial Directional Analysis and Hotspots

We detected the distribution of HIV/AIDS cases among counties using the GIS Spatial Model [12]–[13] (S1). The Global Moran statistic 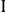
tests for spatial associations of disease at the county level, where 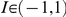. HIV/AIDS cases are clustered, randomly distributed and dispersed when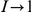, 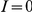 or 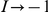, respectively. The standard normal deviate (*z*) was used to test whether cases were distributed randomly. When 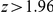 (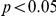), the case was considered to be clustered at the county level.

The Getis 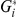
statistic (S1) was used to identify which counties were most likely to be hotspots within a cluster of similar counties. The larger the value of 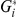
for any county *i*, the greater the influence of that county is. Counties for which 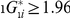 (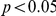) were regarded as hotspots [12]–[13].

The Directional Similarity Based Clustering Method (S1) was used to detect the spatial trend of disease at the national level based on the consistent geographic trend of cluster areas, using counties mapped with ellipse [14].

More detailed information about above spatial analysis method can be found in the Supporting file.

### Multilevel Regression Models

Four 2-level Poisson models (spatial models 1–4) were used to assess factors related to the distributed changes of HIV/AIDS expressed as logarithms, where the country 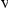 represented the second level and the measurements repeated over time (years) represented the first level. Spatial models 1, 2 and 3 were used to calculate the effect of migrants, length of the highway, and time (years), respectively, on the variation of HIV/AIDS cases among counties. Allowing for the effects of age (0–14, 15–64, and ≥65 years) and gender, the fourth spatial model assessed whether notified cases in counties from 2003 to 2009 were affected by Consumption Expenditure Composition (CEC), defined as Household Consumption Expenditure (total expenditure of resident households on the final consumption of goods and services) / Government Consumption Expenditure (government spending to provide public services to the whole country and net expenditure on goods and services provided by the government to households free of charge or at reduced prices); individual income (per capita annual net income [PCANI] of rural households); urbanization (proportion of urban/rural population at the county level); highway (length at county level); and illiteracy (proportion at county level).

## Results

**Figure 2:**
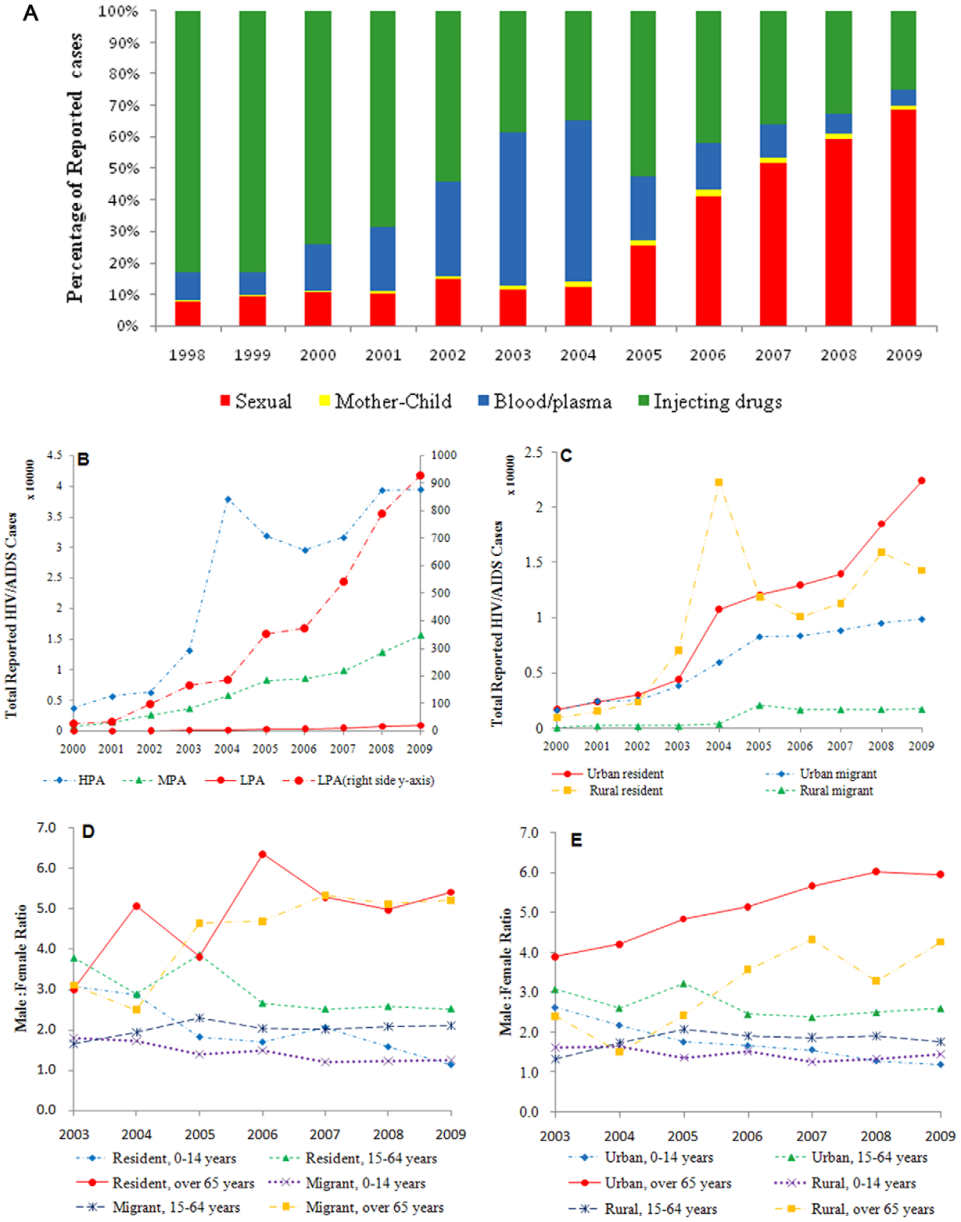
The change of HIV/AIDS among different subpopulations by: (A) percentage of reported HIV/AIDS cases by year and transmission route; (B) total numbers of cases by prevalence regions; (C) total numbers of cases by urban vs. rural and residents vs. migrants; (D) gender ratio by residents vs. migrants, stratified by age and (E) gender ratio by urban vs. rural, stratified by age.

Among 326,157 HIV/AIDS cases in the national HIV/AIDS surveillance system through 2009, 78%, 21% and 1% were reported by HPA, MPA and LPA, respectively; 30% were women, 49% were urban, and 43% were migrants. Between 1989–1994, most reported cases (1191/1426 [83.5%]) were among injecting drug users in Yunnan province. By 1998, all provinces had reported cases, with the proportion infected by routes other than through injecting drugs gradually increasing (Figure 2A).

### Spatial analysis: hotspots and spatial evolution, 2003–2009

**Figure 3:**
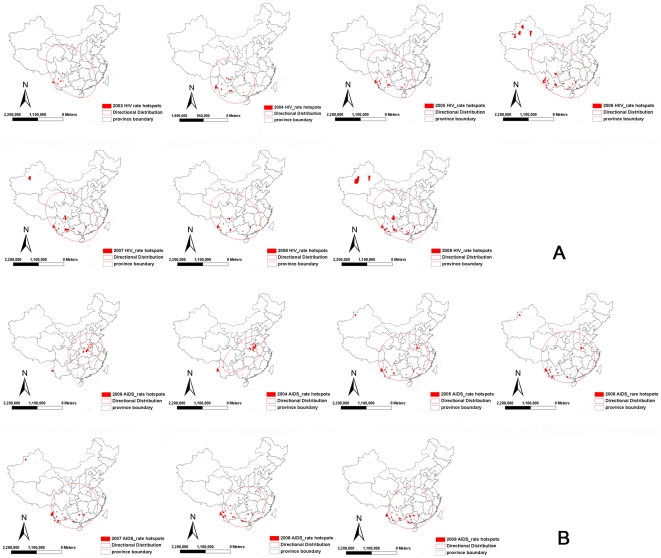
The distribution of hotspots from 2003–2009 by (A) reported HIV cases by year; and (B) reported AIDS cases by year.

From 2003 to 2009, HIV/AIDS cases increased 199% in HPA, 333% in MPA, 459% in LPA (Figure 2B). The values of (*I, z*) [(0.21, 7.9), (0.34, 7.1), (0.45, 17.5), (0.40, 19.1), (0.38, 24.9), (0.45, 27.9) and (0. 51, 34.9)] indicate significant variation and clustering in HIV and AIDS cases among counties across the 7 years, with the differences increasing year by year (Table 1, Model 3). Persistent hotspots of HIV were detected in Guangdong, Guangxi, Henan, Sichuan, and Yunnan Provinces from 2003 to 2005 and in Guangdong, Guangxi, Sichuan, Xinjiang, and Yunnan from 2006 to 2009. The trend evolved along border regions from the South to the Northwest and expanded inwards over time. In contrast, however, AIDS cases were centered in central, eastern China (Henan province) and extended to the Southwest (Figure 3). Cases in both models expanded to the big cities, for example Shanghai and Beijing.

**Table 1:** Factors associated with the spread of HIV/AIDS, 2003–2009.

Parameters	Estimate 95%CI		S.E	χ^2^ value	P value
**Model 1: Impact of time on variance of HIV/AIDS between counties**					
**Fixed Effects**					
Constant	−10.522 (−11.221, −9.823)		0.357	868.678	<0.001
Year	0.076 (0.287, 1.163)		0.067	1.292	0.256
**Random Effects**					
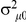	3.618 (1.660, 5.576)		0.999	13.110	<0.005
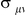	−0.379 (−0.070, 1.257)		0.156	5.885	<0.05
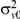	0.095 (0.028, 0.162)		0.034	7.739	<0.05
**Model 2: Impact of migrants on variance of HIV/AIDS between counties**					
**Fixed Effects**					
Constant	−10.391 (−11.286, −9.508)		0.454	524.430	<0.001
Migrant	1.429 (−10.663, 12.429)		5.660	0.064	0.800
**Random Effects**					
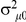	4.507 (0.163, 0.191)		1.578	8.157	<0.001
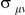	−36.995 (−70.671, −3.320)		17.185	4.634	<0.05
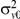	701.335 (225.075, 1177.60)		8.334	243.040	<0.001
**Model 3: Impact of highways on variance of HIV/AIDS between counties**					
**Fixed Effects**					
Constant	−18.283 (−23.024, −13.440)		2.462	5.146	<0.05
Highway	0.725 (0.287, 1.163)		0.225	10.382	<0.005
**Random Effects**					
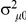	99.202 (14.795, 183.609)		44.905	4.880	<0.05
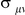	−8.932 (−16.607, 1.257)		4.082	4.634	<0.05
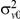	0.819 (0.118,1.521)		0.373	4.821	<0.05
**Model 4: Factors associated with case notification rate of HIV/AIDS**					
**Fixed Effects**					
Constant		−15.950 (−17.133, −14.767)	0.604	679.380	<0.001
Sex		13.010 (11.842, 14.178)	0.596	475.870	<0.001
Age		−8.190 (−8.729, −7.651)	0.275	885.050	<0.001
CEC*		0.021 (0.001, 0.039)	0.010	4.910	<0.05
PCANI†		2.900 (2.752, 3.048)	0.076	466.640	<0.001
Illiterate		0.030 (0.026, 0.034)	0.002	180.020	<0.001
Urbanization‡		−1.090 (−1.232, −0.948)	0.073	223.820	<0.001
Highway§		0.110 (0.097, 0.123)	0.006	300.700	<0.001
Illiterate*PCANI		0.040 (0.024, 0.056)	0.008	30.420	<0.05
Illiterate*Urban		0.060 (0.051, 0.069)	0.005	149.030	<0.001
**Random effects**					
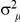		8.110 (4.051, 12.169)	2.215	15.324	<0.05

*CEC: Consumption expenditure composition was defined by household consumption expenditure /government consumption expenditure.

† PCANI: Per Capita Annual Net Income of Rural Households.

‡ Urbanization: urban population/rural population at county level.

§ Highway: the length of highway at county level.

### Case changes in different population groups: 2003−2009

The number of reported HIV/AIDS cases increased among the total population. Compared with 2003, by 2009 there was a 232% increase among the total number of reported HIV/AIDS cases (from 16,862 to 56,027), with a 112% increase among rural residents, 294% increase among urban residents, 211% increase among migrants, and 237% increase among permanent residents (Figure 2B). Of note, there was a spike in reported HIV/AIDS cases in HPA and among rural residents in 2004 (Figure 2B, 2C). The overall increase in permanent residents was more rapid than that in migrants. This difference was found to contribute to the variance of HIV/AIDS between different counties but not within any one county (Figure 2C, Table 1, and Model 1). From 2003 to 2009, the increases were 249% and 219% among men and women, respectively. Younger women (<65) increased more rapidly than younger men but elderly men (≥65) increased more rapidly than elderly women (Figure 2D, 2E). Among migrants and permanent residents, elderly men increased 711% and 2660%, respectively, and women increased 350% and 1536%, respectively (Figure 2D). Similar trends were found in urban and rural areas, with elderly men increasing 1497% and 2121%, respectively, and elderly women increasing 947% and 1150%, respectively (Figure 2E).

### Transmission route among different population groups

Compared with 2003, by 2009 the proportion of HIV infection by sexual transmission increased 504%, whereas the proportion of infection by blood/plasma transmission decreased 90% and by injecting drug user transmission decreased 35% (Figure 2E). Injecting drugs and sexual transmission were the main routes of transmission in HPA (55% and 44%) while sexual transmission predominated in MPA (52%) and LPA (51%). Sexual transmission was also the primary route among permanent residents (33%) while injecting drugs predominated among migrants (39%)(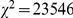,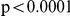). In contrast to rural areas, with concomitant blood/plasma transmission, injecting drug, and sexual transmission patterns (29%, 28%, and 28%, respectively), sexual transmission was the primary transmission route (36%) in urban areas (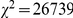 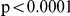). Sexual transmission was also the major route of transmission among women (44%) and the elderly (59% in men, 44% in women), while injecting drugs dominated the 15−64 age group (35%) (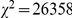,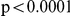).

### Factors associated with HIV/AIDS at the county level

There was significant variation of HIV/AIDS cases among counties (Table 1, Models 1−4), with the trend consistent year by year (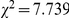,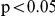; Table 1, Model 1). Migrants (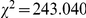,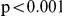) and the length of highway (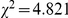,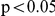) were associated with the distribution of HIV/AIDS among counties (Table 1, Models 2−3). The length of highway was also associated with the HIV/AIDS case notification rate (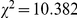,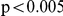) (Table 1, Models 3−4). The labor force population (15−64 years old) (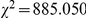,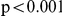), men (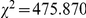,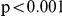), CEC (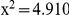,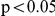), PCANI (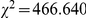,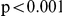) and illiteracy (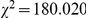,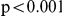) all contributed to HIV/AIDS cases (Table 1, Model 4). Urbanization was associated with reduced HIV/AIDS transmission (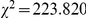,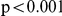). When the interaction between urbanization and illiteracy was examined, the increase in HIV/AIDS associated with illiteracy became even more pronounced (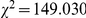,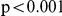) (Table 1, Model 4).

## Discussion

Our study shows that the distribution of HIV/AIDS infection in China is evolving from border regions to inland areas and from the traditional high-risk populations (injecting drug users and sex workers) to the general population (the elderly and women), with changes in the modes of transmission and movement of migrants nationally (Figures 2 and 3, Table 1) [3]. Although HIV/AIDS is believed to have entered China from Yunnan, due to its proximity to the drug routes of the Golden Triangle, examination of the evolving geographical changes of HIV/AIDS demonstrate the spread from border regions to interior regions (Figure 3), consistent with previous molecular epidemiology studies [15]−[16]. This change might be explained by the movement of migrants and an expanded highway network across the country (Table 1, Models 2−4). Initially, most patients were injecting drug users along drug trafficking routes from the Golden Triangle, through southern China, to northwest China (Figure 3), but with the market economic reform and development, many people migrated from rural to urban areas in search of better jobs and a better standard of living. HIV/AIDS was then brought to urban areas through migrants and highways (Table 1, Models 2−3). Our study shows that, from 2003−2009, HIV/AIDS increased in urban residents 2.6 times faster than in rural residents (294% vs. 112%, Figure 2C). In recent years, scattered hotspots of disease have been identified in developed cities, for example Beijing and Shanghai (Figure 3). Migrants contributed to the variance of distribution of HIV/AIDS between counties but were not associated with prevalence within counties (Figure 2C; Table 1, Model 2).

Although migrants contributed to the spread of HIV across the country, reported numbers of HIV/AIDS infections increased in permanent residents more rapidly than in migrants (237% vs. 211%). There are several likely explanations for this. First, the report on China's Migrant Population Development shows that the distribution of migrants among different counties varies [3]. More than 80% of migrants emerge from six provinces (Anhui, Henan, Sichuan, Guizhou, Chongqing and Guangxi) and travel to six provinces (Beijing, Fujian, Guangdong, Jiangsu, Shanghai and Zhejiang), which explains the contribution to variance of HIV/AIDS between counties. Second, although migrants bring HIV/AIDS to the permanent residents in their new location, migrants are a relatively small population overall compared to the permanent residents. Once HIV enters the pool of permanent residents, the potential spread is much greater due to the increased numbers of people. The third reason is the sexual revolution in China [8]−[9]. Several studies have shown that sexual attitudes and behaviors have changed in China, from having one stable sexual partner in traditional marriage to tolerance of multiple sexual partners [17]−[19]. Sexual transmission has become the dominant mode of HIV transmission in China (Figure 2A) and our study shows that sexual transmission is the predominant mode of transmission among permanent residents (33%).

In our analysis, men, the labor force population (15−64 years old) and people in HPA are the major contributors of HIV/AIDS cases (Table 1, Model 4; Figure 2B), which is consistent with the government report about HIV/AIDS in China [10]. However, it is notable that rapid increases in reported HIV/AIDS cases primarily due to sexual transmission have been identified among women (219% increase, 44% by sexual transmission), those ≥65years old (1506% increase, 59% by sexual transmission), MPA (353% increase, 52% by sexual transmission), and LPA (459% increase, 51% by sexual transmission) (Figure 2B, 2D, 2E). This dramatic growth of HIV among women and the elderly is likely due to several reasons. First, the recent predominance of sexual transmission indicates that national HIV transmission patterns have changed (Figure 2A, Figure 3) and HIV is now spreading from the traditional high-risk populations to the general population (Figure 2B, 2D, 2E). There has, in recent years, been a marked increase in the number of sexual partners among women and the elderly, including premarital and extramarital partners [20]. Second, the increased screening of HIV/AIDS among the general population, for example, pre-operation screening in health centers, has identified those HIV-infected among the general population, who tend to be more female and elderly. Third is the interaction of increasing urbanization combined with illiteracy (Table 1, Model 4).

Spikes in the reported numbers of HIV/AIDS cases were noted in 2004 among HPA (Figure 2B) and rural residents (Figure 2C). These spikes were due to the large-scale HIV screening conducted among former plasma donors in and around Henan province [21]. The unique HIV epidemic among former plasma donors in China has already been extensively described [22], with infections occurring around 1994 and the subsequent development of AIDS roughly 8−10 years later. Although this cohort was largely confined to Henan and the adjacent areas of surrounding provinces, the size of this epidemic continues to impact national data. This is demonstrated in Figure 3, where the directional distribution of newly reported HIV cases is towards the northwest and not significantly affected by Henan province because there are relatively few new infections there since plasma donating was banned around 1996 (Figure 3A). In contrast, Henan province represents a significant proportion of newly reported AIDS cases, particularly around 2004 when large-scale HIV screenings were conducted, thus keeping the directional distribution focused in southern-central China (Figure 3B). The spread of AIDS cases towards the northwest is poorly represented partly because infections have not yet progressed to AIDS and also partly because of a reporting bias, with no large scale screening done in areas other than Henan. A large proportion of AIDS patients in other areas likely just have not been identified and reported.

This analysis has a few limitations. First, our analysis is based on reported cases of HIV/AIDS across China and may not be a true representation of the actual spread. However, because this analysis includes 326,157 cases, which are all reported cases of HIV across the entire country, it is still a very powerful analysis with important trends. Second, treatment of HIV/AIDS has been shown to prevent HIV transmission [23]−[25] and certainly has affected the distribution and evolution of HIV/AIDS in China. Although we were not able to include the effect of treatment in our current analysis, other analyses have already demonstrated the impact of HIV treatment on decreasing mortality and improving treatment outcomes in China [26]−[28]. Additional analyses are needed to learn the effect of treatment on the prevention and control of HIV/AIDS in China.

This analysis of changes in the characteristics of reported HIV/AIDS cases over time demonstrates several important trends critical to inform future prevention and control efforts in China. First, HIV likely entered China through rural border regions and subsequently spread throughout the country via migrants. Second, once HIV spread from migrants to local permanent residents, subsequent transmissions between permanent residents and within the general population occurred rapidly and increasingly sexually. The increase in sexual transmissions then resulted in more infections among women and elderly men. The overall numbers of reported HIV/AIDS cases has increased throughout the country. Economic, social and cultural factors have all affected the distribution of HIV/AIDS. To reduce the continued spread of HIV, effective prevention and control measures targeting these trends must be developed and implemented. Such measures will require close collaboration between different departments nationally and even internationally due to the rapid globalization and spread of disease, with transmissions converging more and more towards unsafe sexual behavior.

## Supporting Information

Text S1 Methods: 1. Spatial Statistical Analysis. 2. Directional Similarity Based Clustering Method.
(DOC)
