# Supplementary material for: Tracking the Evolution of HIV/AIDS in China from 1989–2009 to Inform Future Prevention and Control Efforts
Source: PLoS One. 2011 Oct 5;6(10):e25671. doi: 10.1371/journal.pone.0025671 (PMC3187780; doi:10.1371/journal.pone.0025671)
Supplement: Text S1 — Methods: 1. Spatial Statistical Analysis. 2. Directional Similarity Based Clustering Method. (DOC) [file pone.0025671.s001.doc]

**S1:**

**Tracking the Evolution of HIV/AIDS in China from 1985-2009 to Inform Future Prevention and Control Efforts**

**Run title: Evolution of HIV in China**

Zhongwei Jia^1, 2^ (Ph.D), Lu Wang^2^ (Ph.D), Ray Y. Chen^3^, Dongmin Li^2^, Lan Wang^2^, Qianqian Qin^2^, Zhengwei Ding^2^, Guowei Ding^2^, Chunpeng Zang^2^ (M.D), Ning Wang^2^* (M.D)

**1. Spatial Statistical Analysis**

Here we give further details of the Moran and Getis statistics that are used to identify, respectively, clustering and hotspots of reported TB cases. Moran's $I$ is a weighted correlation coefficient used to detect departures of case notification rate from spatial randomness. It tests for spatial autocorrelation of the notifications rates from counties and determines whether the rates of neighboring counties are similar, measuring the spatial patterns of disease with$Z score$. Moran's $I$is defined by

$$I\left(d\right)=\frac{n\sum_{i}^{n} \sum_{j}^{n} w_{ij}\left(d\right)\cdot\left(y_{i}-\bar{y}\right)\left(y_{j}-\bar{y}\right)}{\left(\sum_{i}^{n} \sum_{j}^{n} w_{ij}\left(d\right)\right)\cdot\sum_{i}^{n} \left(y_{j}-\bar{y}\right)^{2}},$$

where $n=1,2...2859$ denote 2859 counties in our study; $y_{i}$ denotes TB case notification rate at county$i$; $\bar{y}$ is the mean value of $\left\{y_{i}\right\}$ over the 2859 counties; $w_{ij}$is the spatial weight measure defined as 1 if county $i$ is contiguous to county$j$ and 0 otherwise; $I\in\left(-1,1\right)$, where a positive value means that nearby counties have similar case notification rates indicating global spatial clustering and negative values indicate that nearby counties have different rates indicating global dispersion.

The$Z score$are used to test the significance of $I$ values, which are used to determine the spatial pattern of disease. At a significance level of$p<0.05$, $Z score$ would have to be less than –1.96 or greater than 1.96, whereupon county case notification rates are significantly dispersed or clustered nationwide. The$Z score$ is defined by:

$$Z score\left(I\right)=\frac{I-E\left(I\right)}{\left[Var\left(I\right)\right]^{1/2}},$$

Getis’$G_{i}^{*}$ statistic measures local spatial autocorrelation and is used to detect counties that are hotspots, among all 2859 counties. Getis’$G_{i}^{*}$ is defined as:

$$G_{i}^{*}=\frac{\sum_{j}^{n} w_{ij}\left(d\right)\cdot y_{i}-W_{i}^{*}\cdot\bar{y}}{S\cdot\left\{\left[nS_{1i}^{*}-W_{i}^{*2}\right]/\left(n-1\right)\right\}^{1/2}},$$

where$n$, $y_{i}$, $\bar{y}$, are as above; $d$ is the threshold value of distance for a hotspot area; $S$ is the standard variance; $\left\{w_{ij}\right\}$is a symmetric binary spatial weighting matrix with$w_{ij}$ = 1 if county$j$is within a given distance $d$ from $i$ and 0 otherwise; $W_{i}^{*}=\sum_{j\neq i}^{n} w_{ij},$ $S_{1i}^{*}=\sum_{j\neq i}^{n} w_{_{ij}}^{2}.$ The higher the value of $G_{i}^{*}$ for county $i$, the greater the influence of that county. The counties with higher rates, which$G_{i}^{*}\geq1.96$, also a$Z score$, are defined as hotspots.

**2. Directional Similarity Based Clustering Method**

The Directional Similarity Based Clustering Method (DSCM) utilizes the objective function based on the directional similarity measure, and then optimizes it using the fixed point iteration method such that all the stable states of the data samples are derived．By presenting all these stable states to the hierarchical clustering algorithm AHC，the final clustering results are obtained．More detail information can be found in reference [1-3].

**Reference**

1. Yang MS, Wu KL. A similarity based robust clustering method [J]．IEEE Trans on Pattern Analysis and Machine Intelligence. 2004: 26; 434—448.
2. Mardia KV, Jupp P．Directional Statistics[M]．2nd edition．New York：John Willey and So ns Ltd，2000.
3. Banerjee A，DhilIon IS，Ghosh J，el a1．Generative model based clustering of directional data [C]．Conference on Knowledge Discovery in Data，Washington，DC, 2003.
